# Salacine—the Surgeon General’s Circular

**Published:** 1845-08

**Authors:** 


The following article is one of much interest to practitioners in miasmatic regions. It is to them of the highest importance to have in their possession a substitute for the salts of quinia. Should the supply, by any accident of war, or otherwise, be cut off, deplorable indeed would be the result. Various species of the willow and poplar, containing salicine, are to be found in almost all sections of our country, and should it be discovered to possess the anti-periodic and febrifuge properties of quinia, the supply could not fail. Every physician should supply himself with salicine, and as opportunity affords, test its virtues. We would be happy to publish the results of such observations, in conducting and preparing the report of which, the circular of the Surgeon General may be taken as the guide.

*Salicine—the Surgeon General's circular.*—It redounds to the general reputation and to the high medical character of our country, that the highest medical officer in the Government is distinguished for his zeal in the profession for which he was educated. In the following circular, issued by him, an ardent desire is manifested for determining an important question; and to accomplish this object, there is a minuteness of detail required in the returns to be made at Washington, which must yield the most satisfactory results. We shall be happy to publish these results, whenever attainable. The following is the circular, signed by Thomas Lawson, Surg. Gen.:

"Sir,—The Medical Purveyor at New York has been directed to issue to those Military Posts, at which miasmatic diseases are of frequent occurrence, a supply of *salicine* (the active principle of the bark of the common *willow*)—a medicine which has been recommended by high authorities for its *febrifuge* and *anti-periodic* virtues.

"Inasmuch as the supply of the *sulphate of quinine* is, at best, precarious, and as, moreover, it may be diminished, at any time, by an interruption of our commercial relations with foreign nations, it becomes the duty of officers of the Government who are intrusted with the health of those engaged in the public service, to use their best endeavors to provide a substitute for a remedy so highly valued, and so universally employed.

"I have therefore deemed it advisable to submit the salicine to trial on a large scale, with a view of ascertaining to what extent it may be relied upon as a substitute for the sulphate of quinine, in a case of emergency, and accordingly I have to request that you will institute a fair and impartial trial of its remedial powers, in your practice, in all cases of miasmatic disease in which the administration of quinine may not be indispensably requisite—and in such other cases as you may think proper.

"You will forward to this office a special report of your observations on the subject, on or before the expiration of the current year, noticing particularly the following points:—

"1. The doses in which you have employed salicine—with their effects.

"2. The diseases, and conditions of the system, in which it has been administered—and with what effect.

"3. Whether you have found it more, or less, liable to irritate the stomach, than sulphate of quinine.

"4. Any bad consequences you may have observed to follow its employment, attributable to the medicine.

"5. Any combinations you may have found to affect its activity; and what preparation of the system you have found necessary before its exhibition.

"6. Your opinion of its *modus operandi.*

"7. Its value as a remedy, as compared with sulphate of quinine, and other medicines of similar properties.

"8. Brief and concise notes of cases in which it has been employed in your practice—as numerous as practicable.

"It is proper to add, that as the profession at large will, doubtless, be interested in the results of these observations, they will probably be given to the public, in such form as will be most creditable to the observers."—*Bost. Med. Jour.*

